# Research note: Antiviral effect of remdesivir against experimental Newcastle disease virus infection in chickens

**DOI:** 10.1016/j.psj.2025.105022

**Published:** 2025-03-11

**Authors:** Hiewa Othman Dyary

**Affiliations:** Department of Basic Sciences, College of Veterinary Medicine, University of Sulaimani, New Sulaimani, Street 27, Sulaymaniyah 46001, Kurdistan Region, Iraq

**Keywords:** *in vivo* study, Newcastle disease virus, mortality rate, broilers

## Abstract

Newcastle disease (ND), caused by the Newcastle disease virus (NDV), has no approved therapy, and vaccination often fails to eradicate infection in endemic areas. In this study, the antiviral effect of remdesivir was tested against experimental ND in broilers. First, the NDV was isolated and identified from an outbreak in a broiler farm in Sulaymaniyah, Iraq. Then, it was propagated in embryonated chicken eggs, and its median embryo lethal dose (ELD_50_) was determined. This ELD_50_ was used to infect broilers when the antiviral effect of remdesivir was tested. Forty-eight Ross-308 broiler chicks were assigned into six equal groups. Group 1 was the negative control and did not undergo any treatment. Group two (positive control) was intranasally infected with NDV when the chicks reached 17 days and left without treatment. Groups 3 to 5 were infected with the virus on day 17 and treated with remdesivir at 2 mg/kg, 4 mg/kg, and 6 mg/kg, respectively. Remdesivir treatment started simultaneous with the experimental infection, and the drug was administered twice daily for seven days through the subcutaneous route at the chest area. Group 6 administered remdesivir at 6 mg/kg twice daily for seven days without infection. The survival rate in the groups treated with 4 mg/kg and 6 mg/kg remdesivir was 100 %, and the chicks were free from the virus after two weeks. Remdesivir inhibits NDV infection in broilers when administered simultaneously with the virus.

## Introduction

Newcastle disease (ND) is a highly contagious worldwide disease of avian species causing high morbidity and mortality rates, and affected birds show respiratory, digestive, and neurologic symptoms with severe immunosuppression (Ul‐Rahman et al., 2022). ND is caused by the Newcastle disease virus (NDV), which belongs to the genus Avulavirus, family Paramyxoviridae.

There is no approved therapy against NDV, and immunization seems to be the primary way of preventing ND in poultry, especially in semi-intensive and intensive systems. Also, hygienic measures such as cleaning and disinfecting utensils, preventing access to wild birds, and promoting the hygiene of farm employees are combined with vaccination programs to control NDV outbreaks (Abdisa and Tagesu, 2017). Several ND vaccines are used to control the infection, which may eventually lead to fewer birds being infected with the virus (Sá e Silva et al., 2016), but the disease still poses a threat to the poultry industry, indicating that vaccination alone cannot eliminate the infection. Also, vaccination provides complete protection only after around 10–14 days, which means it does not control the disease in farms where the virus has already spread. Hence, developing antiviral agents against NDV seems to be the best way to control the disease.

While SPF-ECEs provide a cost-effective model for testing the antiviral effects of different compounds against NDV, similar results may not be obtained when compounds are tested in infected chickens. This is mainly due to the differences in the immunocompetence and pharmacokinetics between ECEs and live chickens (Vargas et al., 2007).

Remdesivir is a broad-spectrum antiviral agent against several viral families, such as SARSCoV and Ebola. It is a prodrug metabolized to the nucleoside triphosphate derivative (active form), which is incorporated by the viral RNA-dependent RNA polymerase, preventing viral RNA synthesis and replication (Gordon et al., 2020). This study was conducted to test the antiviral activity of remdesivir against Newcastle disease in chickens experimentally infected with the virus.

## Materials and methods

### Study design

The study included isolation and identification of NDV from broilers naturally infected with the virus. Then, the virus was propagated in embryonated chicken eggs (ECEs). The collected chorioallantoic fluid from infected ECEs was used to experimentally infect chicks, followed by testing the antiviral effect of remdesivir. The Ethics Committee at the College of Veterinary Medicine, University of Sulaimani, approved the study protocol through the approval number AUP-2024-14.

### Isolation and identification of NDV from naturally infected chickens

Five infected chickens with clinical signs of ND were brought to the Vet Lab Clinic in Sulaimani Province. After postmortem examination, samples from the brain, lungs, cecal tonsils, and tracheal secretions were collected and ground and the viral RNA was extracted using Total RNA Mini Kit for tissues (AddBio-Korea). The extracted RNA was used in polymerase chain reaction (PCR) to confirm infection with ND.

The forward primer 5`-ATGGGCYCCAGACYCTTCTAC-3′ and reverse primer 5′-CTGCCACTGCTAGTTGTGATAATCC-3′ were used to multiply the 535-basepair (bp) NDV Fusion (F) gene fragment (Desingu et al., 2021).

The PCR tube contained 10 µL master mix, 5 µL RNA, 1 µL (10 pmol) of each of the forward and reverse primers, and 3 µL of Q water. The tubes were put in a thermocycler (Prime United Kingdom) that was programmed to run denaturation at 95°C for five minutes, followed by 55 cycles of denaturation at 95°C for 30 s, annealing at 57°C for 30 s, and extension at 72°C for 40 s. A final extension at 72°C for five minutes concluded the PCR.

The PCR products (negative, positive, and test samples) were run on a 1 % agarose gel for 45 min at 135 volts. Then, the SafeBlue Illuminator/Electrophoresis system visualized the PCR product after the gel was illuminated using UV light. A 100-bp ladder was used to compare the bands.

The organs of the diseased chickens were also examined for infectious bronchitis and avian influenza, two common diseases in the region, using PCR, and the results showed that the chickens were clear of these diseases.

### Newcastle disease virus isolation in embryonated chicken eggs

The NDV was isolated in ECEs, and the 50 percent embryo lethal dose (ELD_50_) was calculated. The methodology and results of isolating NDV and ELD_50_ calculations were published previously (Husseiun et al., 2023). The calculated ELD_50_, 6.3 × 10^8^ times dilution of the collected allantoic fluid, was used to infect chickens when the effect of remdesivir was tested *in vivo* against NDV in experimentally infected chickens.

### Effect of remdesivir on NDV

One-day-old chicks (Ross-308 broiler) from nonvaccinated mothers for NDV were brought from a local hatchery. The chicks were raised under standard conditions until they became 17 days old. Then, they were randomly divided into six groups, each with eight chicks. Each group was kept in a 100 cm × 100 cm space, and the floor was covered with corrugated fiberboard. Feed and water were provided ad libitum; commercial broiler feed was purchased from a local factory, and tap water was boiled and let to cool down before being provided to the chicks. The room was air-conditioned, and ventilation was controlled using ventilation fans. The temperature was set according to the standard requirements.

The treatment protocols were as follows: Group 1, the negative control, was not infected with NDV and did not receive treatment. Group 2, the positive control, was infected with NDV-containing allantoic fluid. Groups 3 to 5 were infected with NDV and treated with 2.0 mg/kg, 4.0 mg/kg, and 6.0 mg/kg remdesivir (remdesivir 100 mg/vial (Veklury®) by GILEAD, USA), respectively, twice a day for seven days. Group 6 was given 6.0 mg/kg remdesivir without virus infection. Group 1 and Group 6 were kept in a separate room to avoid infection with NDV. Strict quarantine measures were followed to prevent the virus from spreading to the uninfected chicks (groups 1 and 6) and the environment. Infection with the NDV was through the nasal route with 0.2 mL of the allantoic fluid of infected ECEs after being diluted by 6.3 × 10^8^ times.

Remdesivir treatment started on the same day the chicks were infected with NDV (day 17) by subcutaneous injection in the chest. The dosage of remdesivir has not been determined in birds. Hence, it was extrapolated from the human dosage according to the body surface area by multiplying the human dosage by 3.1 (Nair and Jacob, 2016). The calculated dosage was about 3.9 mg/kg. However, the dosage was approximated to 4.0 mg/kg, and a higher dosage (6.0 mg/kg) and a lower dosage (2.0 mg/kg) were included.

The chicks were observed for the appearance of clinical signs suggestive of ND, and their body temperature was measured using a thermometer. The mortality rates were scored. Also, tracheal and cloacal swab samples were taken for virus detection using PCR, and the same protocol previously used in this study for NDV isolation from a natural infection was followed. The swabs were collected on days 20, 23, 26, 29, 32, and 35. Also, postmortem examination was done on the chicks that did not survive the disease.

### Data analysis

The data of mortality rates were analyzed by descriptive statistics, and the differences between groups were compared using the independent samples Kruskal-Wallis test. A probability value of less than 0.05 was considered significant.

## Results and discussion

The chicks showed no signs of illness until they reached 17 days. They were alert and healthy, with body temperatures within the normal ranges. Feeding and water consumption were within the normal ranges as well.

Remdesivir's bioavailability in chickens has not been established following oral administration, and its first-pass hepatic metabolism is the primary reason for its low bioavailability (Xie and Wang, 2021). Hence, it was injected subcutaneously into the chest area of the treated chicks in this study. Moreover, the dosage rate of remdesivir has not been established in birds. So, the dosage was extrapolated from the human dose according to the body surface area and metabolic rate, as proposed by Nair and Jacob (2016).

After the chicks of four groups (2, 3, 4, 5) were infected with NDV on day 17, the positive control group (Group 2) showed clinical signs suggestive of ND after four days (day 21). The clinical signs included increased body temperature, greenish and white diarrhea, ruffled feathers, increased body temperature, sneezing, gasping, and reduced feed consumption.

The clinical signs of ND appeared on the chicks of Group 3 after five days of infection, but the severity of the signs was less than those of the untreated control group. Also, the clinical signs appeared after six days of infection in groups 4 and 5, but they were less severe than that of Group 3, especially in Group 5 chicks, which were treated with the highest concentration of remdesivir.

Mortalities in the positive control group started after six days (day 23) of infection. Two of the eight chicks (25 %) died on day six postinfection. On the following day, 3/8 (37.5 %) died, and 2/8 (25 %) died on day eight postinfection. By day nine postinfection, all the chicks died in the positive control group. The average mortality rate in this group was 25 % per day. On postmortem examination, the liver was congested, and petechial hemorrhage was evident on the intestines, cecal tonsils, and gland tips in the proventriculus. These lesions are diagnostic of Newcastle disease.

**Fig. 1:**
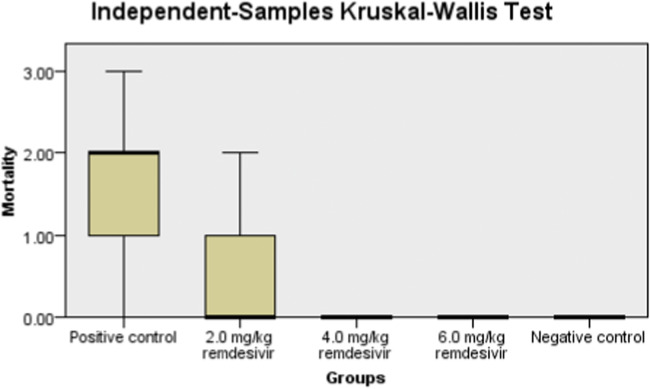
Number of dead chicks per day from day six to day 10 postinfection with NDV.

Two chicks from Group 3 died after eight days of infection, and one chick died after nine days of infection (Table 1), and 62.5 % of the chicks survived the NDV infection in this group. The survival rate from NDV infection in groups 4 and 5 was 100 %, even after 18 days of infection when the chicks were 35 days old. There was a significant difference in the mortality rate among the groups when it was compared by the independent samples Kruskal-Wallis test (*p* = 0.008), indicating that remdesivir remdesivir significantly reduced the mortality rates in all treated groups, compared to the positive control (Fig. 1).

**Table 1:** Mortality rates in the different groups after experimental infection with NDV.

Group	Age of chicks (days)*													Number survived	Cumulative mortality (%)
	23	24	25	26	27	28	29	30	31	32	33	34	35		
1 (negative control)	0	0	0	0	0	0	0	0	0	0	0	0	0	8	0
2 (positive control)	2	2	3	1	0	0	0	0	0	0	0	0	0	0	100.0
3 (2.0 mg/kg remdesivir)	0	0	2	1	0	0	0	0	0	0	0	0	0	5	37.5
4 (4.0 mg/kg remdesivir)	0	0	1	0	0	0	0	0	0	0	0	0	0	7	12.5
5 (6.0 mg/kg remdesivir)	0	0	0	0	0	0	0	0	0	0	0	0	0	8	0
6 (6.0 mg/kg remdesivir)	0	0	0	0	0	0	0	0	0	0	0	0	0	8	0

⁎ The chicks were infected with NDV on day 17, but mortalities started on day 23 in the untreated positive control group. Each group comprised eight chicks.

This study reported the potential antiviral effect of remdesivir against experimental infection of broilers with an isolated velogenic NDV from clinical disease in poultry farms. The NDV caused 100 % mortality in the untreated (positive control) chicks, confirming that the virus was a velogenic strain, as reported in a previous study by our team (Husseiun et al., 2023). At the same time, martality was reduced dose-dependent in the treated groups with remdesivir, and treated birds were cleared from the virus after nine days of infection and remained alive for the rest of the experiment, showing that this drug possesses antiviral activity against the virus. As judged by the findings, remdesivir has the potential to reduce the severity of clinical signs and mortality rates due to ND when it is administered to susceptible birds before clinical signs appear.

Remdesivir treatment reduced mortality rates dose-dependently, as 62.5 % of the chicks treated with 2 mg/kg of remdesivir survived the infection, while all chicks treated with 4 mg/kg and 6 mg/kg remdesivir survived. These results showed that remdesivir drastically reduced the mortality rates and severity of infection in the experimentally infected chicks with NDV. Remdesivir is a broad-spectrum antiviral agent against many RNA viruses, and it was approved by the United States Food and Drug Administration (FDA) for the treatment of coronavirus disease 2019 (COVID-19) (Blair, 2023). However, its antiviral activity against NDV has not been reported.

The PCR analysis of the tracheal and cloacal swabs showed that all chicks from the positive control were infected with NDV after three days of infection (day 20), and they remained carriers until all of them died on day 26. Groups 1 and 6 (not infected with NDV) were clear of the virus till the end of the study. NDV was not detected in groups 3, 4, and 5 on day 20, but the virus was detected on days 23 and 26. On day 29 and afterward, all remdesivir-treated groups were clear of the virus until the end of the study. This outcome confirmed that remdesivir therapy successfully inhibited ND in experimentally infected broilers.

In conclusion, remdesivir protects broilers from experimental ND by reducing the mortality rate dose-dependently. Based on this study's outcomes, remdesivir shows potential to control NDV. However, further studies are needed to evaluate its effect against natural infections of NDV and other paramyxoviruses.

## Animals rights statement

The Animal Care and Use Committee at the College of Veterinary Medicine, University of Sulaimani, approved the study protocol through approval number AUP-2023-4.

## Financial disclosure statement

This study received no financial aid.

## Declaration of competing interest

The author declares that there is no conflict of interest to disclose.
